# Rhombic calcite microcrystals as a textural proxy for meteoric diagenesis

**DOI:** 10.1038/s41598-021-04219-2

**Published:** 2022-01-07

**Authors:** Mohammed S. Hashim, Stephen E. Kaczmarek

**Affiliations:** Department of Geological and Environmental Sciences, Western Michigan University, Kalamazoo, MI 49008 USA

**Keywords:** Carbon cycle, Marine chemistry

## Abstract

Numerous Phanerozoic limestones are comprised of diagenetic calcite microcrystals formed during mineralogical stabilization of metastable carbonate sediments. Previous laboratory experiments show that calcite microcrystals crystallizing under conditions similar to those that characterize meteoric diagenetic settings (impurity-free, low degree of supersaturation, high fluid:solid ratio) exhibit the rhombic form/morphology, whereas calcite microcrystals crystallizing under conditions similar to those that prevail in marine and marine burial diagenetic settings (impurity-rich, high degree of supersaturation, low fluid:solid ratio) exhibit non-rhombic forms. Based on these experimental observations, it is proposed here that rhombic calcite microcrystals form exclusively in meteoric environments. This hypothesis is tested using new and previously published textural and geochemical data from the rock record. These data show that the vast majority of Phanerozoic limestones characterized by rhombic microcrystals also exhibit petrographic and/or geochemical evidence (depleted δ^13^C, δ^18^O, and trace elements) indicative of meteoric diagenesis whereas non-rhombic forms are associated with marine burial conditions. By linking calcite microcrystal textures to specific diagenetic environments, our observations bring clarity to the conditions under which the various microcrystal textures form. Furthermore, the hypothesis that rhombic calcite microcrystals form exclusively in meteoric environments implies that this crystal form may be a useful textural proxy for meteoric diagenesis.

## Introduction

Many Phanerozoic (541 Mya to present) limestones are characterized by low-Mg calcite (calcite) microcrystals that typically measure between 1 and 9 µm in diameter and comprise both carbonate matrix and allochems^1^. There is a general consensus that these calcite microcrystals are diagenetic (post-depositional) in origin and form during mineralogical stabilization of metastable aragonite and high-Mg calcite sediments. Stabilization is a coupled reaction that involves the dissolution of metastable minerals accompanied by the precipitation of calcite^2,3^.

**Figure 1:**
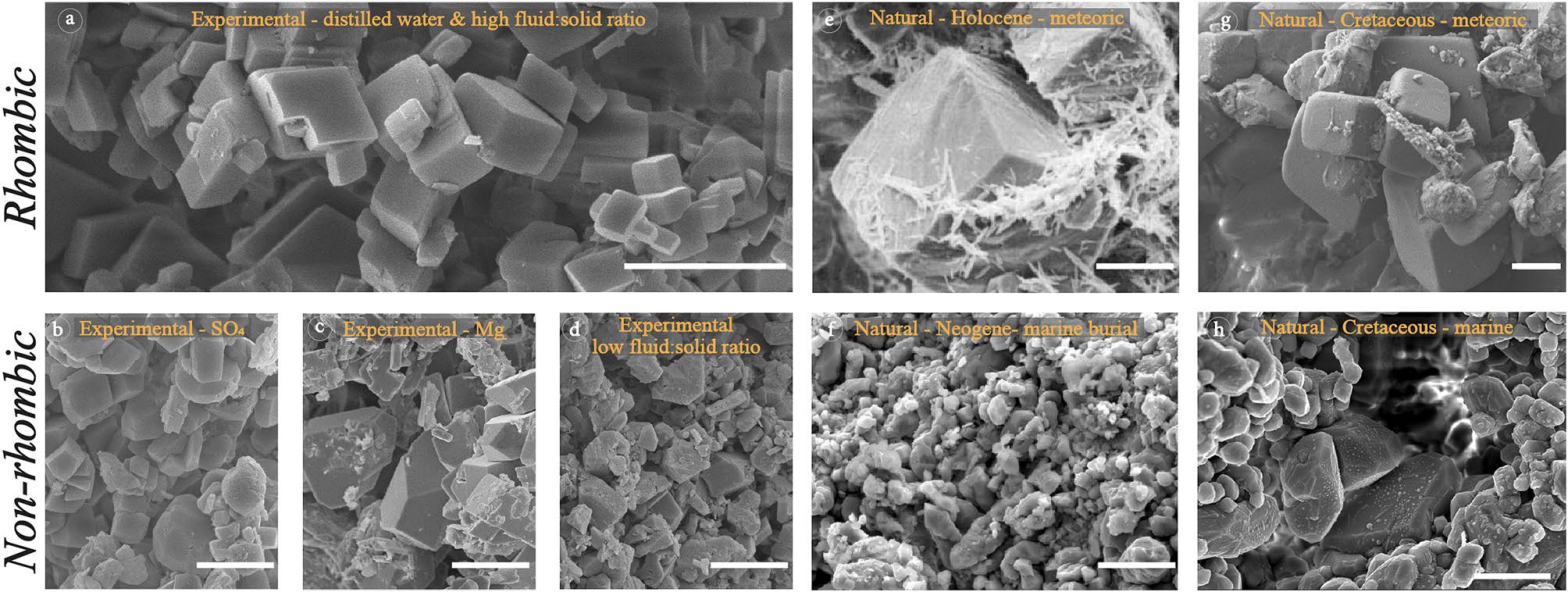
SEM images showing calcite microcrystals from various experimental and natural samples. (**a**) Rhombic microcrystals from aragonite to calcite stabilization experiments in distilled water and high fluid to solid ratio. (**b**) Polyhedral (multi-faceted) microcrystals from stabilization experiments in the presence of SO_4_^2−^ ([Na_2_SO_4_] = 28 mM). (**c**) Polyhedral microcrystals from stabilization experiments in the presence of Mg^2+^ ([MgCl_2_] = 5 mM). (**d**) Polyhedral and anhedral microcrystals from stabilization experiments at low fluid to solid ratio (0.3 mL/g) (**d** is modified after Ref.^16^). (**e**) Rhombic calcite microcrystals among aragonitic needles in Holocene sediments from the Bahamas, interpreted to have crystallized from freshwater (modified after Ref.^21^). (**f**) Polyhedral and anhedral calcite microcrystals from Clino well (1769 ft) drilled on the western edge of the Great Bahama Bank, interpreted to have crystallized from marine-derived pore fluids (modified after Ref.^25^). (**g**) Rhombic calcite microcrystals from the Lower Cretaceous Stuart City Trend interpreted to have formed during meteoric diagenesis. (**h**) Polyhedral calcite microcrystals from a carbonate reservoir in the Middle East interpreted to have formed during marine burial diagenesis. All scale bars are 5 µm.

It is well documented that calcite microcrystals exhibit a wide range of crystal forms/morphologies including rhombic, polyhedral (multi-faceted), rounded, anhedral, and scalenohedral^1,4–9^. The vast majority of calcite microcrystals in Phanerozoic limestones are non-rhombic^1^, and exhibit geochemical signatures compatible with crystallization in marine-derived pore fluids during shallow burial^10^. Laboratory experiments show that calcite microcrystals crystallizing from impurity-free solutions that are slightly supersaturated with respect to calcite (near-equilibrium) are rhombic^11–15^ (Fig. 1a). Similarly, experiments whereby aragonite is stabilized to calcite in distilled water yields rhombic microcrystals^16^. In contrast, calcite microcrystals exhibit non-rhombic forms (scalenohedral, polyhedral, anhedral, etc.) when crystallization occurs in solutions that are highly supersaturated^11^, with high a_Ca_^2+^/a_CO3_^2−^ (a is activity) or high pH^11,17^, or in solutions containing various organic and inorganic impurities^12,14^. Likewise, calcite stabilized from aragonite in fluids containing Mg^2+^, SO_4_^2−^, or excess Ca^2+^, or under low fluid:solid ratios exhibits polyhedral (non-rhombic) and anhedral microcrystals^16^ (Fig. 1b–d).

Based on these experimental observations, we propose that the far more uncommon rhombic calcite microcrystals in Phanerozoic limestones form exclusively in meteoric settings because fluids here are generally devoid of the conditions that have been shown to produce non-rhombic forms. Meteoric diagenetic environments are generally characterized by (i) fluids with much lower [Mg], [Ca], and [SO_4_] compared to seawater^18,19^, (ii) active oxidation of organic matter by O_2_ and SO_4_^2−^ (Refs.^19,20^), (iii) fluids that are undersaturated or are only slightly supersaturated with respect to calcite^18^, and (iv) high fluid to solid (i.e., water/rock) ratios^16^. Using a global compilation of new and previously published textural and geochemical data from Phanerozoic limestones, this study aims to test the hypothesis that rhombic calcite microcrystals form exclusively in meteoric diagenetic environments.

## Results

**Figure 2:**
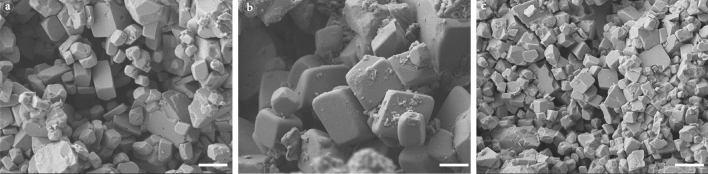
SEM images of rhombic calcite microcrystals from (**a**) Malacca limestone, (**b**) Stuart City Trend, and (**c**) Thamama Gp. Note that Malacca and Stuart City samples are characterized by rhombic microcrystals whereas Thamama sample is characterized by a mixture of rhombic and polyhedral crystals. All scale bars are 5 µm.

**Figure 3:**
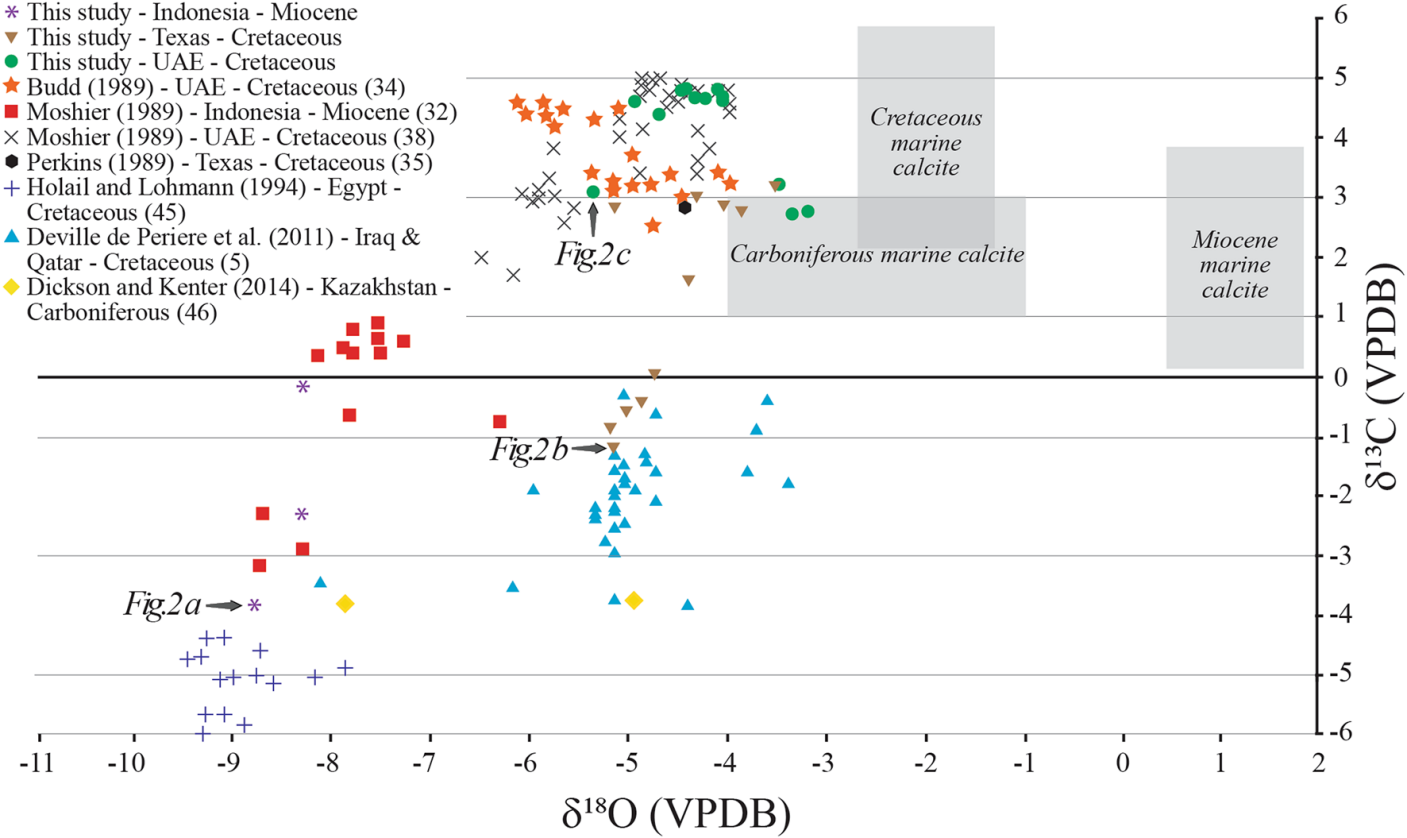
δ^13^C and δ^18^O from Phanerozoic limestones characterized by rhombic calcite microcrystals (Table 1). Gray boxes represent the estimated isotopic composition of marine calcite adopted from Ref.^10^. Gray arrows point to data from samples whose SEM images are shown in Fig. 2.

Of the 31 microcrystalline limestone studies that investigated calcite microcrystals and interpreted their diagenetic origin (Supplementary Table S1), 11 report rhombic calcite microcrystals (Table 1). New and previously published stable isotope data from the limestones with rhombic microcrystals are reported in Fig. 3. δ^13^C measurements range from − 6 to + 5‰ and δ^18^O range from − 9.40 to − 3.20‰ VPDB (Fig. 3). The three limestone units characterized by rhombic calcite microcrystals examined here (Fig. 2) exhibit depleted δ^18^O values, and except for the Thamama Gp. calcites, exhibit depleted δ^13^C values compared to the isotopic composition of age-equivalent marine calcites (Fig. 3). SEM images of natural and laboratory synthesized calcite microcrystals are reported in Fig. 1.

**Table 1:** Studies that report rhombic calcite microcrystals in Phanerozoic limestones.

Study (reference)	Geologic Unit	Age	Location	Interpretation of Diagenetic environment
This study	Malacca Limestone, Stuart City Trend, and Shuaiba Fm	Miocene and Cretaceous	Indonesia; Texas, USA; UAE	Meteoric based on depleted δ^13^C and δ^18^O (except δ^13^C in Shuaiba Fm.)
Steinen 1982^21^	Modern sediments	Holocene	Bahamas	Meteoric based on the observation that the sediments are residing in meteoric water
Moshier 1989^32^	Malacca Limestone	Miocene	Indonesia	Meteoric based on depleted δ^13^C and δ^18^O
da Silva et al. 2009^27^	Campo	Mid-Paleocene	Spain	Meteoric based on exposure surfaces and petrographic observations
Perkins 1989^35^	Stuart City Trend	Cretaceous	Texas, USA	Meteoric or marine burial based on depleted δ^18^O isotopes and trace elements
Loucks et al. 2013^33^	Stuart City Trend	Cretaceous	Texas, USA	No specific environment was inferred
Loucks et al. 2017^44^	Calvin and Winn carbonates	Cretaceous	Louisiana, USA	Meteoric or shallow marine based on petrographic observations
Budd 1989^34^	Thamama Gp	Cretaceous	UAE	Meteoric based on low concentration of trace elements and depleted δ^18^O
Moshier 1989^38^	Thamama Gp	Cretaceous	UAE	Marine burial based on normal δ^13^C and depleted δ^18^O
Holail and Lohmann 1994^45^	Bahariya Oasis Chalks	Cretaceous	Egypt	Meteoric based on depleted δ^13^C and δ^18^O, low concertation of trace elements, and petrographic observations
Deville de Periere et al. 2011^5^	Mishrif Fm	Cretaceous	Iraq; Qatar	Meteoric based on depleted δ^13^C and δ^18^O, and petrographic observations (clay-filled karstic features, dissolution molds, sparry cement), and proximity to an exposure surface
Dickson and Kenter 2014^46^	Bashkirian and Visean	Carboniferous	Kazakhstan	Meteoric based on depleted δ^13^C and δ^18^O and petrographic observations

Representative SEM images from the three limestones studied here are reported in Fig. 2. Textural and isotopic data for all the studied samples are summarized in Supplementary Table S2 and Fig. S1. We examined 45 samples, of which 13 were examined both texturally and isotopically: 3 from Malacca limestone, 5 from Stuart City Trend, and 5 from Thamama Gp. The results from the three Malacca limestone samples show that the dominant calcite microcrystal form is rhombic (Fig. 2a), though polyhedral crystals are also observed, and that all three samples exhibit depleted isotopic values (Supplementary Table S2; Fig. S1). It is also observed that the sample with the most negative δ^13^C is dominated by rhombic crystals whereas the sample with the least negative δ^13^C exhibits a mixture of rhombic and polyhedral crystals (Supplementary Table S2). Similarly, all 5 samples from Stuart City Trend are characterized by either rhombic crystals only, or a mixture of rhombic and non-rhombic (Fig. 2b; Supplementary Fig. S1). Four of the 5 samples exhibit depleted isotopes (Supplementary Table S2). All 5 samples from Thamama Gp. (Shuaiba Fm. and Kharaib Fm.) have enriched δ^13^C values and 4 are characterized by non-rhombic microcrystals (Supplementary Table S2). Only one sample is characterized by unequivocal non-rhombic forms (Supplementary Fig. S1).

## Discussion

Based on numerous published experimental observations (Table 2) demonstrating that rhombic calcite microcrystals form under a set of specific conditions, it is proposed here that the presence of rhombic microcrystals is indicative of formation in meteoric fluids. The key lines of evidence in support of this hypothesis are discussed below, and include observations from laboratory experiments, observations from relatively modern carbonate sediments, and observations from the ancient rock record.

**Table 2:** Observations from previous experimental studies showing the conditions under which rhombic and non-rhombic calcite microcrystals form.

Study (reference)	Type of experiment	Fluid chemistry ([Ca]/[CO_3_])	Saturation state wrt calcite (Ω)	Fluid:solid ratio (mL/g)	Interpreted cause for non-rhombic form	Crystal form
Carmona et al. 2003^11^	Carbonation of Ca(OH)_2_	Ca(OH)_2_, CO_2_ (1.06–1.23)	–	–	–	Rhombic
Kim et al. 2017^15^	Direct precipitation	CaCl_2_, (NH_4_)2CO_3_	–	–	–	
Hashim and Kaczmarek 2020^16^	Aragonite to calcite stabilization	Distilled water	Near equilibrium	150	–	
Davis et al. 2000^12^	Seeded precipitation	NaHCO_3_, CaCl_2_, MgCl_2_,	Near equilibrium	–	Aqueous Mg	Non-rhombic
Carmona et al. 2003^11^	Carbonation of Ca(OH)_2_	Ca(OH)_2_, CO_2_ (> 1.28)	Far from equilibrium	–	High supersaturation and nonstoichiometry (i.e., [Ca]/[CO_3_])	
Ruiz-Agudo et al. 2011^17^	Seeded and non-seeded precipitation	NaHCO_3_, CaCl_2_, NaCl	6.5		High pH	
Kim et al. 2016^14^	Direct precipitation	CaCl_2_, (NH_4_)2CO_3_, glycine and aspartic amino acids	–	–	Amino acids	
Kim et al. 2017^15^	Direct precipitation	CaCl_2_, (NH_4_)2CO_3_, MgCl_2_, polystyrene sulfonate	–	–	Aqueous Mg and polystyrene sulfonate	
Hashim and Kaczmarek 2020^16^	Aragonite to calcite stabilization	NaCl, Na_2_SO_4_	Near equilibrium	150	Aqueous SO_4_	
Hashim and Kaczmarek 2021^3^	Aragonite to calcite stabilization	MgCl_2_, CaCl_2_, CO_2_	Near equilibrium (1–1.5)	0.3	Aqueous Mg	
Hashim and Kaczmarek 2020^16^	Aragonite to calcite stabilization	Distilled water	Near equilibrium	0.8	Low fluid/solid ratio	

### Calcite structural and growth forms

A large body of experimental observations (Table 2) demonstrates that calcite microcrystals crystallizing under conditions similar to those that characterize meteoric settings (impurity-free, low degree of supersaturation with respect to calcite, high fluid:solid ratio) exhibit the rhombic form^11–16^ (Fig. 1a). In contrast, calcite microcrystals crystallizing under experimental conditions similar to those that characterize marine or marine burial settings (impurity-rich, high supersaturation, low fluid:solid ratio) exhibit non-rhombic forms^11,12,14,16,17^ (Fig. 1b–d). Given that crystal form is dictated by the internal crystal structure and the external growth conditions^13^, these experimental observations imply that the rhombic form is the structural form of calcite, and is dictated by its rhombohedral crystal system, whereas non-rhombic forms are growth forms, and are the result of various growth conditions. That is, the absence of the growth conditions that interfere with the growing calcite microcrystals in meteoric settings allows them to achieve their preferred structural rhombic form, and the prevalence of those growth conditions in marine and marine burial settings leads to the various non-rhombic growth forms.

The physical and chemical conditions of crystallization may dictate crystal forms in various ways. Impurities, such as Mg^2+^, have been shown to adsorb on calcite growth steps, altering their orientation, and thus changing calcite microcrystal form from rhombic to elongated, multi-faceted crystals^12,22^. Similarly, the occlusion of some organic additives, such as amino acids, into the calcite lattice has been shown to distort the crystal form by creating heterogenous strain throughout the lattice^14^. In the case of fluids with a high degree of supersaturation and high a_Ca_^2+^/a_CO3_^2−^, the observed alteration in crystal form from rhombic to non-rhombic has been attributed to disproportionate growth rates on the various crystal faces^11^. In contrast, the change in crystal form from rhombic to non-rhombic in high pH fluids has been attributed to the presence of OH^−^ ions which may stabilize the polar scalenohedral crystal faces^17^. Several of the factors that influence crystal form of calcite crystallized directly from a solution have also been shown to influence calcite microcrystals stabilized from aragonite^16^. In addition to the aforementioned factors, stabilization experiments have shown that lowering fluid/solid ratio (solution volume/aragonite reactant mass) leads to the formation of polyhedral and anhedral calcite microcrystals rather than euhedral rhombic crystals, an observation attributed to the increased competition for space among the growing microcrystals^16^.

### Linking calcite microcrystal form to diagenetic environments

The hypothesis that rhombic microcrystals form during meteoric diagenesis is consistent with direct observations from Holocene carbonate sediments currently undergoing stabilization to rhombic calcite in a freshwater setting in the Bahamas^21^ (Fig. 1e), and calcite microcrystals crystallizing from freshwater in various modern environments^22^. In contrast, polyhedral (non-rhombic) calcites in Neogene sediments are interpreted to have formed via stabilization in the marine realm based on stable isotope values that reflect marine fluids^23–25^ (Fig. 1f). The consistency of these observations with the proposed hypothesis is critical since these observations are from relatively modern sediments that have not been buried substantially, which makes the interpretation of their diagenetic history relatively straightforward.

The experimental studies highlight numerous growth conditions that interfere with calcite crystal growth and lead to the development of non-rhombic forms. In natural settings, these specific physical and chemical conditions are commonly encountered by marine carbonate sediments and thus represent the diagenetic rule rather than the exception. For example, aqueous Mg and SO_4_, both shown experimentally to interfere with calcite crystal form, are the most common divalent ions in seawater^26^. Furthermore, marine sediments are more likely to undergo diagenesis in the marine and marine-burial setting, and less likely to experience meteoric diagenesis. Accordingly, if rhombic calcite microcrystals form exclusively during meteoric diagenesis, they would be expected to be rarer, and indeed are, based on the published textural and geochemical data. Supplementary Table S1 indicates that the vast majority of calcite microcrystals are reported to be non-rhombic and are interpreted to form during burial diagenesis from marine fluids based on stratigraphic and geochemical data. Statistics from the published global studies show that ~ 90% of calcite microcrystals in Phanerozoic limestones exhibit non-rhombic forms whereas only 10% are rhombic^1^. Interestingly, nearly 93% of published case studies report geochemical evidence from calcite microcrystals consistent with the marine burial, whereas the remaining 7% report geochemical evidence indicative of meteoric diagenesis^10^. To emphasize, the vast majority of calcite microcrystals in Phanerozoic limestones spanning a wide range of geologic ages, depositional environments, and burial depths, exhibit non-rhombic forms^1^ and their geochemistry indicates formation in the marine burial diagenetic realm^10^.

Phanerozoic limestones characterized by the far more uncommon rhombic calcite microcrystals (Table 1; Fig. 2) often exhibit sedimentologic and petrographic evidence indicative of meteoric diagenesis, such as paleosols, exposure surfaces, and clay-filled karstic cavities^5,27^, and geochemical evidence, such as low trace element concentrations and depleted stable isotope values (Fig. 3). More specifically, the Malacca limestone, Stuart City, and Thamama Gp. limestones, which are characterized by rhombic microcrystals (Fig. 2), all exhibit stratigraphic and sedimentological evidence of an associated unconformity^28–31^, petrographic evidence of one or more exposure surfaces and meteoric diagenesis, such as vuggy porosity and meniscus cement^29,32,33^, and geochemical evidence suggestive of meteoric fluids such as low trace element contents and stable isotope values depleted relative to associated marine deposits^32,34,35^.

Stable carbon and oxygen isotopes are routinely used to interpret the diagenetic fluids of crystallization of carbonate minerals. Depletion in both δ^13^C and δ^18^O with respect to the inferred isotopic signature of age-equivalent marine calcites, for example, results in what has been called the “inverted J trend” for meteoric diagenesis^10,36,37^. As illustrated in Fig. 3, this isotopic trend characterizes all but one of the case studies that report rhombic calcite microcrystals (Table 1) and measure their stable isotopes (Fig. 3). Moreover, two of the three limestone units examined here (Malacca and Stuart City Trend) exhibit the inverted J trend. In these examples, the samples with the most depleted δ^13^C values exhibit rhombic calcite microcrystals, whereas samples with less depleted or enriched δ^13^C values are dominated by non-rhombic calcite microcrystals (Supplementary Table S2 and Fig. S1). Collectively, these observations are consistent with the proposed hypothesis that rhombic calcite microcrystals form in meteoric fluids.

The Thamama Gp., which is dominated by non-rhombic calcite microcrystals but also contains occasional rhombic calcite microcrystals (Fig. 1c; Supplementary Fig. S1), although generally consistent with the proposed hypothesis, reveals a more complicated story. All the 22 Thamama Gp. samples examined here texturally are characterized by non-rhombic microcrystals except one sample characterized by unequivocal rhombic microcrystals and two others dominated by rhombic with a few non-rhombic calcite microcrystals (Supplementary Table S2 and Fig. S1). Thamama Gp. limestones have low trace element concentrations^34,38^, depleted δ^18^O, but seemingly normal marine δ^13^C (Fig. 3), which Moshier (1989) used to argue that the calcite microcrystals formed via diagenetic stabilization of a metastable CaCO_3_ precursor in the marine burial realm. He reasoned that the δ^13^C reflected marine fluids and suggested that the low Sr concentrations (< 200 ppm) in the calcite microcrystals reflects either a low-Sr, calcite-rich precursor sediment, or multiple episodes of recrystallization in an open system. Using similar data, Budd (1989) interpreted the calcite microcrystals in the Thamama to reflect diagenetic stabilization in meteoric fluids based on the depleted δ^18^O and trace elements (Mg, Sr, Fe, and Mn). He argued that although depleted δ^13^C is common in carbonates crystallized in meteoric settings, isotopic depletion would be insignificant if the fluid:solid ratio was low (i.e., rock buffered), or if the organic carbon content was low in sediments, as is typically the case in an arid setting^34^.

While the diagenetic environment responsible for the calcite microcrystals in Thamama Gp. has been debated, the presence of rhombic calcite microcrystals (Fig. 3c) likely indicates that Thamama Gp. limestones must have been exposed to meteoric fluids at some point during diagenesis. That being said, the dominance of non-rhombic calcite forms in the Thamama (Supplementary Fig. S1) implies that the exposure to meteoric diagenesis was perhaps limited or it was preceded and/or followed by diagenesis in marine burial settings, which may also explain the positive δ^13^C values. Based on field, petrographic, sedimentological, stratigraphic, and geochemical data from Oman [south of the area studied here and by Budd (1989) and Moshier (1989)], Rameil et al. (2012) concluded that multiple transient subaerial exposures preceded and followed by marine hardground stages may have occurred near the top Shuaiba (i.e., top Thamama Gp.) as a result of a number of low-amplitude, high-frequency sea level changes. Importantly, they noted that sea level oscillations may have destroyed the unequivocal evidence for meteoric diagenesis expected from such a spatially (> 100,000 km^2^) and temporally (up to 10 Myr) substantial discontinuity. This interpretation attests to the complex history of Thamama Gp. on the Arabian platform and may explain the more ambiguous isotopic (Fig. 3) and textural data (Supplementary Fig. S1) in the Thamama Gp. limestones, which may simply indicate an ephemeral meteoric influence. In summary, the Malacca and Stuart City Trend limestones are both dominated by rhombic microcrystals and show clear geochemical evidence for meteoric diagenesis, whereas Thamama Gp. limestones are dominated by a mixture of non-rhombic and rarer rhombic calcite microcrystals and exhibit ambiguous geochemical signals suggestive of intermittent exposure to meteoric and marine fluids, or perhaps geochemical and textural resetting during later diagenesis.

### Textural obliteration by late diagenesis

Despite data suggesting that rhombic calcite microcrystals exclusively form during meteoric diagenesis, meteoric fluids may not always produce rhombic microcrystals given the multitude of diagenetic factors that can interfere with the form of the growing crystals. For example, a few case studies reported calcite microcrystals with a mixture of rhombic and non-rhombic forms and suggested, based on petrographic and geochemical evidence, that they have formed during meteoric diagenesis^39,40^ or during either meteoric or marine diagenesis^4,41^. Assuming the calcite microcrystals described in these studies formed in the meteoric realm, several hypotheses can explain why the microcrystals did not become perfectly or only rhombic. For example, in one case study^39^, the microcrystals were observed on surfaces and interiors of ooids, which have been shown experimentally to stabilize to polyhedral calcite microcrystals rather than rhombic, despite stabilization occurring in distilled water, possibly due to the presence of organic matter^16^. It is also not unreasonable to suspect that some rhombic calcite microcrystals initially formed in meteoric settings are altered by later diagenesis, thus obscuring their original crystal form. For example, partial dissolution of rhombic calcite microcrystals during burial diagenesis has been proposed to produce rounded, polyhedral microcrystals^4^, a phenomenon that was recently documented in laboratory experiments^42^. Textural evidence of dissolution, such as rounded edges and inter-crystal gulfs, was noted in the Stuart City Trend limestones (sample 7 in Supplementary Fig. S1). It has also been suggested that compaction and cementation during burial can alter calcite crystal form^4,5,9^. Therefore, later diagenesis may obliterate the initial morphological signature imparted by meteoric diagenesis, similar to geochemical resetting^37^, thus complicating the use of calcite microcrystal form as a proxy for diagenetic fluids.

### Implications and the path forward

The hypothesis that rhombic calcite microcrystals form exclusively during meteoric diagenesis has several implications. Given that calcite microcrystals host the vast majority of limestone microporosity^1,2^, understanding their diagenetic history allows for more accurate prediction of microporosity. The proposed relationship between calcite microcrystal form and diagenetic environment further provides an independent line of evidence to interpret the sedimentary rock record. Textural criterion for the meteoric environment is especially desirable given the equivocality of stable isotope and trace element data in differentiating between diagenetic environments^20,34,37,38^. Notably, 12 out of 31 (39%) of the studies examined here (Supplementary Table S1) invoke more than one diagenetic environment for the origin of calcite microcrystals based on isotope and trace element data, attesting to the inconclusiveness of these proxies. Lastly, the hypothesis that rhombic calcite microcrystals form exclusively in meteoric settings implies that their chemical signatures do not reflect marine conditions and thus they are not suitable for palaeoceanographic reconstructions.

The proposed hypothesis is supported by several lines of evidence from laboratory experiments, modern sediments, and the ancient rock record. However, because the diagenetic history of carbonates is often complicated, it is challenging to pinpoint specific diagenetic environments based on bulk isotopic measurements. Accordingly, future work could further test the proposed hypothesis using microanalytical techniques, which have the potential to reveal a more detailed history of the calcite microcrystals^43^. Future studies should also employ other geochemical tools such as trace elements and clumped isotopes that may be less equivocal in decoding diagenesis than traditional stable isotopes to further test the proposed hypothesis.

## Conclusions

Textural and geochemical data compiled here suggest that the rhombic calcite microcrystals observed in Phanerozoic limestones most likely form via mineralogical stabilization of a metastable CaCO_3_ precursor during meteoric diagenesis. This interpretation is based in part on experiments showing that calcite microcrystals formed under the chemical and physical conditions similar to those of meteoric settings are rhombic, whereas calcite microcrystals formed under conditions similar to those of marine burial settings are non-rhombic. The hypothesis that rhombic calcite microcrystals form exclusively in meteoric environments is further supported by observations from carbonate sediments in relatively modern environments and observations from the ancient rock record. Collectively, these observations imply that rhombic calcite microcrystals may be a useful textural proxy for limestone diagenesis in meteoric settings.

## Methods

Textural and geochemical data from 31 studies of microcrystalline limestones are compiled in Supplementary Table S1. New textural observations and stable isotope data from three of these, the Miocene Malacca Limestone (Belumai Fm.), Indonesia^25,28,29^, Cretaceous Stuart City Trend, Texas, USA^30^, and Cretaceous Thamama Gp., UAE^14,25^, are reported in Supplementary Table S2 and Figs. 2 and 3. In this study, we examined 3 samples from Malacca limestone (Belumai Fm.) from two wells drilled in the North Sumatra Basin, Indonesia, 11 samples from Stuart City Trend from two wells drilled in Pawnee Field, Texas, U.S.A., and 31 samples from Thamama Gp. from 5 wells from UAE (Supplementary Table S2). Some of the samples were characterized both texturally and isotopically. In those cases, we sampled small rock chips from cores and performed a thorough textural analysis. We then powered a small proportion of those same texturally well characterized chips for isotopic analysis.

Imaging was performed on a JEOL 7500 Field Emission SEM using an accelerating voltage of 5 kV and a working distance of 6 ± 0.2 mm. Samples were coated with 10 nm of osmium. Microcrystals are defined as crystals with a diameter between 1 and 10 µm^1^. Rhombic refers to euhedral rhombohedral crystals with 6 rhombuses, whereas polyhedral refers to multi-faceted crystals having > 6 faces. The terms euhedral, subhedral, and anhedral refer to crystals with well-defined, moderately defined, and poorly defined crystal faces, respectively. Stable carbon and oxygen isotopes were measured by placing ~ 10 µg of pure carbonate samples in a stainless steel boats. Samples were then reacted at 77 ± 1 °C with anhydrous phosphoric acid in a Finnigan MAT Kiel IV preparation device coupled directly to the inlet of a Finnigan MAT 253 triple collector isotope ratio mass spectrometer. O^17^ corrected data were corrected for acid fractionation and source mixing by calibration to a best-fit regression line defined by NBS 18 and NBS 19 standards. Data are reported in ‰ notation relative to VPDB. Analytical precision is maintained at better than 0.1‰ for both δ^13^C and δ^18^O.

## Supplementary Information

Supplementary Information.
