# Supplementary material for: Rhombic calcite microcrystals as a textural proxy for meteoric diagenesis
Source: Sci Rep. 2022 Jan 7;12:213. doi: 10.1038/s41598-021-04219-2 (PMC8742114; doi:10.1038/s41598-021-04219-2)
Supplement: Supplementary file 1 — Supplementary Information. [file 41598_2021_4219_MOESM1_ESM.pdf]

Supplementary Material for:

Rhombic Calcite Microcrystals as a Textural Proxy for Meteoric Diagenesis

Mohammed S. Hashim[1 *], and Stephen E. Kaczmarek[1]

[1]Department of Geological and Environmental Sciences, Western Michigan University, Kalamazoo, MI 49008, USA

Supplementary Table S1. A list of studies that reported calcite microcrystals in Phanerozoic marine limestones and interpreted their diagenetic origin.

Reference	Age	Location	Crystal Form	Diagenetic Environment	Evidence*†
Steinen (1982)	Holocene	Bahamas	Rhombic	Meteoric	Currently residing in meteoric fluids
Steinen (1978)	Pleistocene	Barbados	Polyhedral	Meteoric or mixing	Currently residing in meteoric fluids
Saller and Moore (1989)	Pleistocene and Oligocene	Enewetak Atoll, Marshall Islands	Polyhedral	Marine burial	$\delta^{13}C$ is similar to marine sediments, $\delta^{18}O$ is compatible with precipitation from seawater at cool temperature (~ 15 °C)
Lucia (2017)	Miocene–Pleistocene	Bahamas	Polyhedral; anhedral	Marine burial	-
Lucia and Loucks (2013)	Miocene–Pleistocene	Bahamas	Polyhedral; anhedral	Marine burial	-
Melim et al. (2002)	Neogene	Bahamas	Polyhedral; anhedral	Seafloor, marine burial, deep burial	$\delta^{13}C$ and $\delta^{18}O$ are both positive
Lasemi and Sandberg (1984)	Oligocene–Pleistocene	Florida, U.S.A., Bahamas, Mexico, Italy	Polyhedral	Meteoric	-
Loucks et al. (1998)	Eocene	Tunisia	Polyhedral; aligned crystals;	Shallow and deep marine burial	Petrographic observations
da Silva et al. (2009)	Paleocene	Spain	Rhombic; polyhedral	Meteoric	Petrographic observations (exposure surface, paleosols)
Valencia and Laya (2020)	Oligocene - Miocene	Venezuela	Polyhedral	Shallow marine	Petrographic observations, paragenesis,
Moshier (1989a)	Miocene	Indonesia	Rhombic	Meteoric	Depleted $\delta^{13}C$ and $\delta^{18}O$
	Paleocene, Cretaceous	Iran, Libya, Arabian Gulf	Polyhedral	Meteoric or marine	-
Moshier (1989b)	Cretaceous	U.A.E.	Polyhedral; rhombic	Marine	$\delta^{13}C$ values similar to the inferred marine signature, depleted $\delta^{18}O$ compatible with formation and elevated temperature (burial).
Budd (1989)	Cretaceous	U.A.E.	Polyhedral; rhombic	Meteoric	Depleted $\delta^{18}O$ compatible with meteoric fluids, low concentration of trace elements (Mg, Sr, Mn, Fe) compatible with open system diagenesis
Cox et al. (2010)	Cretaceous	U.A.E.	Polyhedral	Marine burial	Depleted $\delta^{18}O$ interpreted to reflect burial temperatures
Deville de Periere et al. (2011)	Cretaceous	Iraq, U.A.E, Qatar	Polyhedral; anhedral; elongated; rhombic	Meteoric and Marine	Petrographic observations (exposure surface, clay-filled karastic features, cements, moldic porosity), depleted $\delta^{13}C$ and $\delta^{18}O$ and compatible with freshwater
Dewever et al. (2007)	Cretaceous	Albania	Polyhedral	Marine burial	Positive $\delta^{13}C$ and slightly negative $\delta^{18}O$ interpreted to reflect seawater derived fluids
Holail and Lohmann (1994)	Cretaceous	Egypt	Rhombic	Meteoric	Depleted $\delta^{13}C$ and $\delta^{18}O$ and low concentration of trace elements (Na and Sr)
			Anhedral; polyhedral	Marine	Positive $\delta^{13}C$ and slightly negative $\delta^{18}O$ and high concentration of trace elements
Loucks et al. (2017)	Cretaceous	Louisiana, U.S.A.	Rhombic	Meteoric and/or shallow marine	Petrographic observations
Perkins (1989)	Cretaceous	Texas, U.S.A.	Rhombic; polyhedral	Meteoric or deep marine	Depleted $\delta^{18}O$ values consistent with meteoric fluids or marine waters at higher temperature, and low trace element concentrations support open system diagenesis

Loucks et al. (2013)	Cretaceous	Texas, U.S.A.	Rhombic; polyhedral	Meteoric and/or shallow marine	Petrographic observations (pendulous cement, wholesale aragonite dissolution)
Richard et al. (2007)	Cretaceous	France	Subhedral rhombic; polyhedral	Meteoric	Depleted $\delta^{13}C$ and $\delta^{18}O$, and low trace element concentrations
Lambert et al. (2006)	Jurassic & Cretaceous	Iraq, U.A.E.	Polyhedral; rounded	Meteoric or deep marine	Positive $\delta^{13}C$ and negative $\delta^{18}O$ values
Volery et al. (2010b)	Jurassic & Cretaceous	France	Subhedral rhombic; polyhedral	Meteoric	Depleted $\delta^{13}C$ and $\delta^{18}O$, low concentrations of Sr and Mg, and petrographic observations
Carpentier et al. (2015)	Jurassic	France	Polyhedral with some rhombic	Meteoric and marine burial	Positive $\delta^{13}C$ and negative $\delta^{18}O$ compatible with precipitation from meteoric fluids or marine fluids at higher temperature
Ahr (1989)	Jurassic	Texas, U.S.A.	Polyhedral; anhedral	Meteoric	Positive $\delta^{13}C$ and negative $\delta^{18}O$, trace elements (Mg, Fe, Mn, Sr, Na, Al, Zn, S), and petrographic observations,
Dravis (1989)	Jurassic	Texas, U.S.A.	Subhedral rhombic	Deep burial	Positive $\delta^{13}C$ and negative $\delta^{18}O$, and petrographic observations
Tavakoli and Jamalian (2018)	Permian & Cretaceous	Arabian Gulf	Subhedral rhombic; polyhedral	Meteoric	-
Dickson and Kenter (2014)	Devonian – Carboniferous	The Caspian Sea, Kazakhstan	Rhombic; polyhedral; anhedral	Meteoric (but marine was not excluded)	Depleted $\delta^{13}C$ and $\delta^{18}O$, petrographic observations, and CL petrography
Kaldi (1989)	Devonian	Canada	Subhedral; Rhombic; polyhedral; aligned crystals	Meteoric or shallow marine	Petrographic observations
Al-Aasm and Azmy (1996)	Devonian	Canada	Polyhedral; anhedral; aligned crystals	Shallow or intermediate burial in modified marine water	Positive $\delta^{13}C$ and very negative $\delta^{18}O$ interpreted to reflect formation at higher temperature during burial. Trace elements (Mg, Mn, Sr, Na) Petrographic observations (e.g., wholesale dissolution)
Munnecke et al. (1997)	Silurian & Pliocene	Bahamas, Sweden	Polyhedral	Shallow marine burial	Positive $\delta^{13}C$ and $\delta^{18}O$, and petrographic observations

* $\delta^{13}C$ and $\delta^{18}O$ refer to carbon and oxygen isotopes in carbonates relative to VPDB.

† Depletion is relative to the age-equivalent isotopic composition of marine calcite.

Supplementary Table S2. A summary of the textural and stable isotopic data for all samples examined in this study.

Sample no.*	SEM Image no.	Geologic unit	Well	Age	Location	Crystal Form	$\delta^{13}C$ (VPDB)	$\delta^{18}O$ (VPDB)
1	1-1	Mallaca	A2	Miocene	Indonesia	Rhombic	-2.33	-8.29
1	1-2	Mallaca	A2	Miocene	Indonesia	Rhombic	-	-
1	1-3	Mallaca	A2	Miocene	Indonesia	Rhombic	-	-
1	1-4	Mallaca	A2	Miocene	Indonesia	Non-rhombic	-	-
1	1-5	Mallaca	A2	Miocene	Indonesia	Mixed rhombic and non-rhombic	-	-
2	2-1	Mallaca	A2	Miocene	Indonesia	Mixed rhombic and non-rhombic	-0.15	-8.30
2	2-2	Mallaca	A2	Miocene	Indonesia	Polyhedral	-	-
2	2-3	Mallaca	A2	Miocene	Indonesia	Polyhedral	-	-
2	2-4	Mallaca	A2	Miocene	Indonesia	Polyhedral	-	-
2	2-5	Mallaca	A2	Miocene	Indonesia	Polyhedral	-	-
2	2-6	Mallaca	A2	Miocene	Indonesia	Polyhedral	-	-
2	2-7	Mallaca	A2	Miocene	Indonesia	Polyhedral	-	-
3	3-1	Mallaca	A3	Miocene	Indonesia	Mixed rhombic and non-rhombic	-3.80	-8.76
3	3-2	Mallaca	A3	Miocene	Indonesia	Mixed rhombic and non-rhombic	-	-
3	3-3	Mallaca	A3	Miocene	Indonesia	Mixed rhombic and non-rhombic	-	-
3	3-4	Mallaca	A3	Miocene	Indonesia	Rhombic	-	-
3	3-5	Mallaca	A3	Miocene	Indonesia	Rhombic	-	-
3	3-6	Mallaca	A3	Miocene	Indonesia	Rhombic	-	-
3	3-7	Mallaca	A3	Miocene	Indonesia	Rhombic	-	-
3	3-8	Mallaca	A3	Miocene	Indonesia	Rhombic	-	-
3	3-9	Mallaca	A3	Miocene	Indonesia	Rhombic	-	-
3	3-10	Mallaca	A3	Miocene	Indonesia	Rhombic	-	-
3	3-11	Mallaca	A3	Miocene	Indonesia	Rhombic	-	-
4	4-1	Stuart City Trend	Schroder2	Cretaceous	Texas, U.S.A.	Rhombic	-0.58	-5.01
4	4-2	Stuart City Trend	Schroder2	Cretaceous	Texas, U.S.A.	Mixed rhombic and non-rhombic	-	-
	4-3	Stuart City Trend	Schroder2	Cretaceous	Texas, U.S.A.	Mixed rhombic and non-rhombic	-	-
5	5-1	Stuart City Trend	Schroder2	Cretaceous	Texas, U.S.A.	Rhombic/rounded	0.04	-4.75
5	5-2	Stuart City Trend	Schroder2	Cretaceous	Texas, U.S.A.	Polyhedral	-	-
6	6-1	Stuart City Trend	Krause4	Cretaceous	Texas, U.S.A.	Rhombic	-1.18	-5.15
6	6-1	Stuart City Trend	Krause4	Cretaceous	Texas, U.S.A.	Rhombic	-	-
6	6-2	Stuart City Trend	Krause4	Cretaceous	Texas, U.S.A.	Rhombic	-	-
6	6-3	Stuart City Trend	Krause4	Cretaceous	Texas, U.S.A.	Rhombic	-	-
6	6-4	Stuart City Trend	Krause4	Cretaceous	Texas, U.S.A.	Rhombic	-	-
6	6-5	Stuart City Trend	Krause4	Cretaceous	Texas, U.S.A.	Rhombic	-	-
6	6-6	Stuart City Trend	Krause4	Cretaceous	Texas, U.S.A.	Rhombic	-	-
6	6-7	Stuart City Trend	Krause4	Cretaceous	Texas, U.S.A.	Rhombic	-	-
6	6-8	Stuart City Trend	Krause4	Cretaceous	Texas, U.S.A.	Rhombic	-	-
6	6-9	Stuart City Trend	Krause4	Cretaceous	Texas, U.S.A.	Rhombic	-	-
7	7-1	Stuart City Trend	Schroder2	Cretaceous	Texas, U.S.A.	Mixed rhombic and non-rhombic	1.60	-4.40
7	7-2	Stuart City Trend	Schroder2	Cretaceous	Texas, U.S.A.	Rhombic/rounded	-	-
8	8-1	Stuart City Trend	Krause4	Cretaceous	Texas, U.S.A.	Rhombic	2.88	-5.15
8	8-2	Stuart City Trend	Krause4	Cretaceous	Texas, U.S.A.	Mixed rhombic and non-rhombic	-	-
8	8-3	Stuart City Trend	Krause4	Cretaceous	Texas, U.S.A.	Mixed rhombic and non-rhombic	-	-
9	9-1	Thamama Gp.	Sajaa-3	Cretaceous	U.A.E.	Polyhedral	4.79	-4.48
9	9-2	Thamama Gp.	Sajaa-3	Cretaceous	U.A.E.	Polyhedral	-	-
9	9-3	Thamama Gp.	Sajaa-3	Cretaceous	U.A.E.	Polyhedral	-	-
10	10-1	Thamama Gp.	Sajaa-3	Cretaceous	U.A.E.	Polyhedral	3.22	-3.48
10	10-2	Thamama Gp.	Sajaa-3	Cretaceous	U.A.E.	Polyhedral	-	-
11	11-1	Thamama Gp.	Sajaa-3	Cretaceous	U.A.E.	Polyhedral	2.72	-3.35
11	11-2	Thamama Gp.	Sajaa-3	Cretaceous	U.A.E.	Polyhedral	-	-
11	11-3	Thamama Gp.	Sajaa-3	Cretaceous	U.A.E.	Polyhedral	-	-
12	12-1	Thamama Gp.	Sajaa-3	Cretaceous	U.A.E.	Polyhedral	4.65	-4.06
12	12-2	Thamama Gp.	Sajaa-3	Cretaceous	U.A.E.	Polyhedral	-	-
12	12-3	Thamama Gp.	Sajaa-3	Cretaceous	U.A.E.	Polyhedral	-	-

12	12-4	Thamama Gp.	Sajaa-3	Cretaceous	U.A.E.	Polyhedral	-	-
13	13-1	Thamama Gp.	M5	Cretaceous	U.A.E.	Polyhedral	-	-
14	14-1	Thamama Gp.	M5	Cretaceous	U.A.E.	Polyhedral	-	-
14	14-2	Thamama Gp.	M5	Cretaceous	U.A.E.	Polyhedral	-	-
14	14-3	Thamama Gp.	M5	Cretaceous	U.A.E.	Polyhedral	-	-
15	15-1	Thamama Gp.	M6	Cretaceous	U.A.E.	Polyhedral	-	-
15	15-2	Thamama Gp.	M6	Cretaceous	U.A.E.	Polyhedral	-	-
15	15-3	Thamama Gp.	M6	Cretaceous	U.A.E.	Polyhedral	-	-
15	15-4	Thamama Gp.	M6	Cretaceous	U.A.E.	Polyhedral with some rhombic	-	-
15	15-5	Thamama Gp.	M6	Cretaceous	U.A.E.	Polyhedral	-	-
16	16-1	Thamama Gp.	M6	Cretaceous	U.A.E.	Rhombic	3.08	-5.28
16	16-2	Thamama Gp.	M6	Cretaceous	U.A.E.	Rhombic	-	-
16	16-3	Thamama Gp.	M6	Cretaceous	U.A.E.	Rhombic	-	-
16	16-4	Thamama Gp.	M6	Cretaceous	U.A.E.	Rhombic	-	-
16	16-5	Thamama Gp.	M6	Cretaceous	U.A.E.	Rhombic	-	-
17	17-1	Thamama Gp.	M7	Cretaceous	U.A.E.	Polyhedral	-	-
17	17-2	Thamama Gp.	M7	Cretaceous	U.A.E.	Polyhedral	-	-
18	18-1	Thamama Gp.	M4	Cretaceous	U.A.E.	Polyhedral	-	-
18	18-2	Thamama Gp.	M4	Cretaceous	U.A.E.	Polyhedral	-	-
19	19-1	Thamama Gp.	M4	Cretaceous	U.A.E.	Polyhedral with a few rhombic	-	-
19	19-2	Thamama Gp.	M4	Cretaceous	U.A.E.	Polyhedral	-	-
20	20-1	Thamama Gp.	M4	Cretaceous	U.A.E.	Polyhedral	-	-
20	20-2	Thamama Gp.	M4	Cretaceous	U.A.E.	Polyhedral	-	-
21	21-1	Thamama Gp.	M4	Cretaceous	U.A.E.	Polyhedral	-	-
22	22-1	Thamama Gp.	M4	Cretaceous	U.A.E.	Polyhedral	-	-
23	23-1	Thamama Gp.	M4	Cretaceous	U.A.E.	Polyhedral	-	-
24	24-1	Thamama Gp.	M4	Cretaceous	U.A.E.	Polyhedral	-	-
25	25-1	Thamama Gp.	M4	Cretaceous	U.A.E.	Polyhedral	-	-
26	26-1	Thamama Gp.	M4	Cretaceous	U.A.E.	Polyhedral	-	-
27	27-1	Thamama Gp.	M4	Cretaceous	U.A.E.	Polyhedral	-	-
28	28-1	Thamama Gp.	M4	Cretaceous	U.A.E.	Polyhedral	-	-
29	29-1	Thamama Gp.	M4	Cretaceous	U.A.E.	Polyhedral	-	-
30	30-1	Thamama Gp.	M4	Cretaceous	U.A.E.	Polyhedral	-	-
31	-	Stuart City Trend	Schroder2	Cretaceous	Texas, U.S.A.	-	-0.52	-4.98
32	-	Stuart City Trend	Schroder2	Cretaceous	Texas, U.S.A.	-	-0.84	-5.10
33	-	Stuart City Trend	Schroder2	Cretaceous	Texas, U.S.A.	-	2.75	-3.87
34	-	Stuart City Trend	Schroder2	Cretaceous	Texas, U.S.A.	-	2.89	-4.05
35	-	Stuart City Trend	Schroder2	Cretaceous	Texas, U.S.A.	-	3.18	-3.52
36	-	Stuart City Trend	Krause4	Cretaceous	Texas, U.S.A.	-	3.03	-4.31
37	-	Thamama Gp.	Sajaa-3	Cretaceous	U.A.E.	-	4.39	-4.70
38	-	Thamama Gp.	Sajaa-3	Cretaceous	U.A.E.	-	4.69	-4.06
39	-	Thamama Gp.	Sajaa-3	Cretaceous	U.A.E.	-	4.82	-4.11
40	-	Thamama Gp.	Sajaa-3	Cretaceous	U.A.E.	-	4.82	-4.44
41	-	Thamama Gp.	Sajaa-3	Cretaceous	U.A.E.	-	4.68	-4.34
43	-	Thamama Gp.	Sajaa-3	Cretaceous	U.A.E.	-	4.61	-4.95
44	-	Thamama Gp.	Sajaa-3	Cretaceous	U.A.E.	-	4.66	-4.23
45	-	Thamama Gp.	Sajaa-3	Cretaceous	U.A.E.	-	2.77	-3.20

* Samples 1 - 12 and 16 were examined texturally and analyzed isotopically. Samples 13 - 30 were examined only texturally and their isotopes are reported in previous studies (Table S1 and Fig. 3). Samples 31 - 45 were analyzed only for their isotopic compositions and their textures are reported in previous studies (Table S1). See Figure S1 below for the textural data of sample 1 - 30.

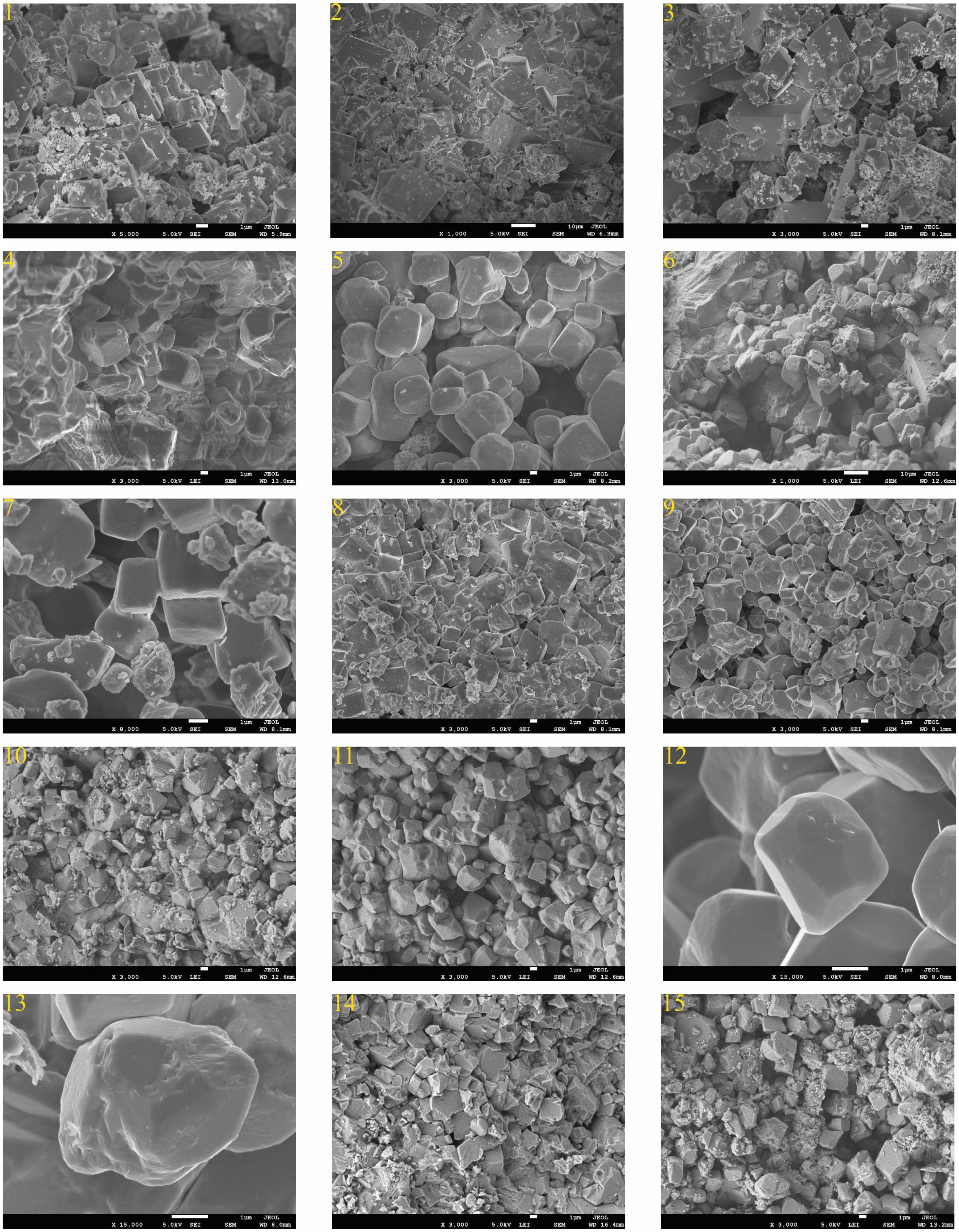
1
2
3
4
5
6
7
8
9
10
11
12
13
14
15

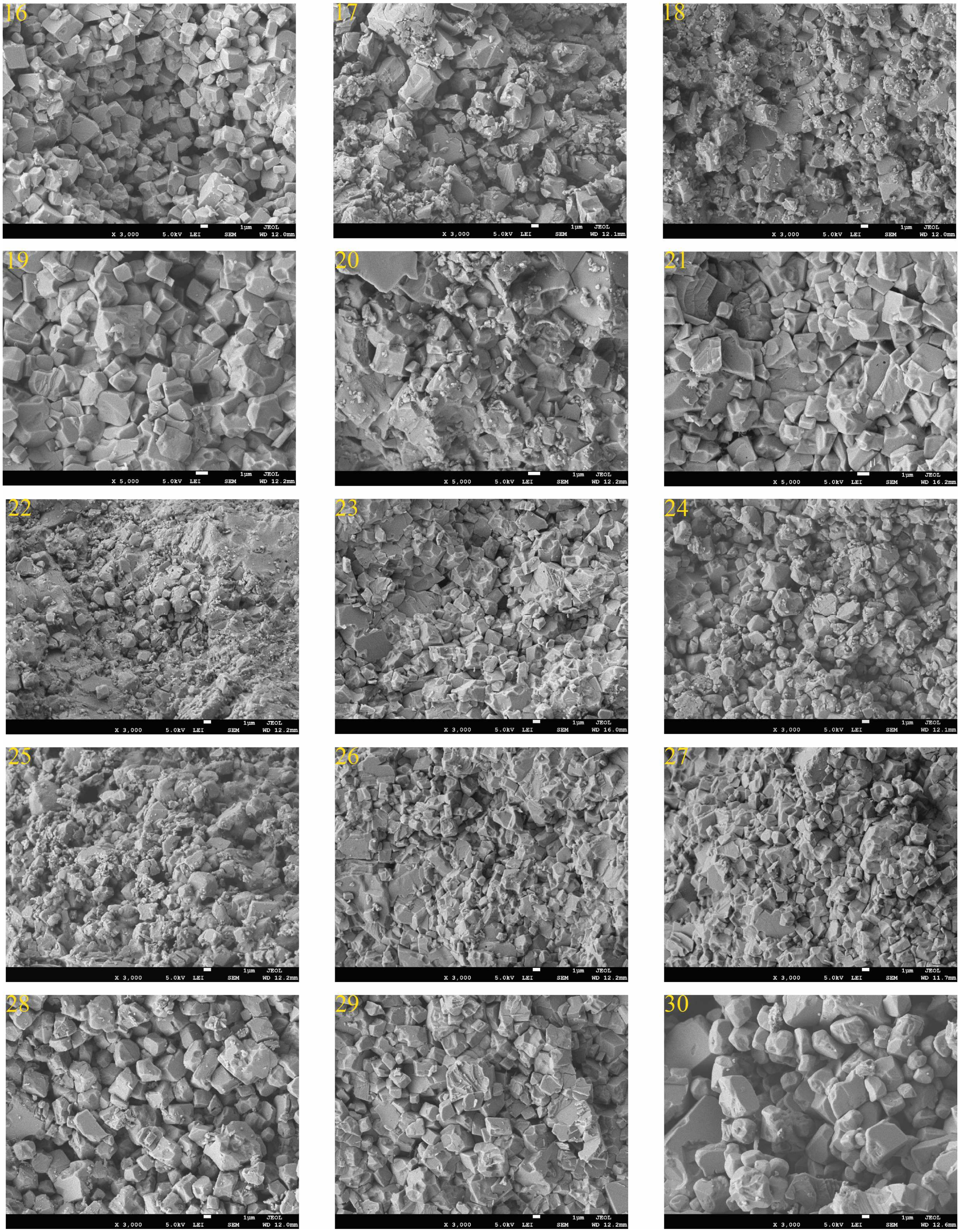

Supplementary Figure S1. SEM images for all the samples examined in this study (samples 1 - 30 in Table S2 above). Textural interpretations (rhombic, non-rhombic) and the isotopic values for each sample are listed in Table S2.

References

Steinen, R.P., 1978. On the diagenesis of lime mud; scanning electron microscopic observations of subsurface material from Barbados, WI. Journal of Sedimentary Research, 48(4), pp.1139-1148.

Steinen, R.P., 1982, SEM observations on the replacement of Bahamian aragonitic mud by calcite: Geology, v. 10, p. 471-475.

Saller, A.H., and Moore, C.H., Jr, 1989, Meteoric diagenesis, marine diagenesis, and microporosity in Pleistocene and Oligocene limestones, Enewetak Atoll, Marshall Islands: Sedimentary Geology, v. 63, p. 253–272, doi: 10.1016/0037-0738(89)90135-8.

Lucia, F.J. and Loucks, R.G., 2013, Micropores in carbonate mud: early development and petrophysics, GCAGS Journal, v. 2, p. 1-10.

Lucia, F.J., 2017. Observations on the origin of micrite crystals. Marine and Petroleum Geology, 86, pp.823-833.

Melim, L.A., Westphal, H., Swart, P.K., Eberli, G.P. and Munnecke, A., 2002. Questioning carbonate diagenetic paradigms: evidence from the Neogene of the Bahamas. Marine Geology, 185(1-2), pp.27-53.

Loucks, R.G., Moody, R.T.J., Bellis, J.K., and Brown, A.A., 1998, Regional depositional setting and pore network systems of the El Garia Formation (Metlaoui Group, Lower Eocene), offshore Tunisia: Geological Society, London, Special Publications, v. 132, p. 355–374, doi: 10.1144/GSL.SP.1998.132.01.20.

Lasemi, Z. and Sandberg, P.A., 1984. Transformation of aragonite-dominated lime muds to microcrystalline limestones. Geology, 12(7), pp.420-423.

da Silva, A.C., Loisy, C., Cerepi, A., Toullec, R., Kiefer, E., Humbert, L., Razin, P., 2009. Variations in stratigraphic and reservoir properties adjacent to Mid-Paleocene sequence boundary, Campo section, Pyrenees, Spain. Sedimentary Geology 219, 237e251.

Valencia, F.L. and Laya, J.C., 2020. Deep-burial dissolution in an Oligocene-Miocene giant carbonate reservoir (Perla Limestone), Gulf of Venezuela Basin: Implications on microporosity development. Marine and Petroleum Geology, 113, p.104144.

Moshier, S.O., 1989a. Microporosity in micritic limestones: a review. Sedimentary geology, 63(3-4), pp.191-213.

Moshier, S.O., 1989b, Development of microporosity in a micritic limestone reservoir, Lower Cretaceous, Middle East: Sedimentary Geology, v. 63, p. 217–240, doi: 10.1016/0037-0738(89)90133-4.

Budd, D.A., 1989, Micro-rhombic calcite and microporosity in limestones: a geochemical study of the Lower Cretaceous Thamama Group, UAE: Sedimentary Geology, v. 63, p. 293–311, doi: 10.1016/0037-0738(89)90137-1.

Cox, P.A., Wood, R.A., Dickson, J.A.D., Rougha, Al, H.B., Shebl, H., and Corbett, P.W.M., 2010, Dynamics of cementation in response to oil charge: Evidence from a Cretaceous carbonate field, U.A.E.: Sedimentary Geology, v. 228, p. 246–254, doi: 10.1016/j.sedgeo.2010.04.016.

Deville de Periere, M., Durlet, C., Vennin, E., Lambert, L., Caline, B., Bourillot, R., and Poli, E., 2011, Morphometry of micrite particles in cretaceous microporous limestones of the Middle East: Influence on reservoir properties: Marine and Petroleum Geology, v. 28, p. 1727–1750, doi: 10.1016/j.marpetgeo.2011.05.002.

Dewever, B., Breesch, L., Mezini, A. and Swennen, R., 2007. Sedimentological and marine eogenetic control on porosity distribution in Upper Cretaceous carbonate turbidites (central Albania). Sedimentology, 54(2), pp.243-264.

Holail, H., and Lohmann, K.C., 1994, The role of early lithification in development of chalky porosity in calcitic micrites: Upper Cretaceous chalks, Egypt: Sedimentary Geology, v. 88, p. 193–200, doi: 10.1016/0037-0738(94)90061-2.

Loucks, R.G., Lucia, F.J. and Waite, L.E., 2013, Origin and description of the micropore network within the lower Cretaceous Stuart City Trend tight-gas limestone reservoir in Pawnee Field in South Texas, GCAGS Journal, v. 2, p. 29-41

Perkins, R.D., 1989, Origin of micro-rhombic calcite matrix within Cretaceous reservoir rock, West Stuart City Trend, Texas: Sedimentary Geology, v. 63, p. 313–321, doi: 10.1016/0037-0738(89)90138-3.

Richard, J, Sizun, J.P., and Machhour, L., 2007, Development and compartmentalization of chalky carbonate reservoirs: the Urgonian Jura-Bas Dauphiné platform model (Génissiat, southeastern France): Sedimentary Geology v. 198, p. 195-207.

Volery, C., Davaud, E., Durlet, C., Clavel, B., Charollais, J., and Caline, B., 2010b, Microporous and tight limestones in the Urgonian Formation (late Hauterivian to early Aptian) of the French Jura Mountains: Focus on the factors controlling the formation of microporous facies: Sedimentary Geology, v. 230, p. 21–34, doi: 10.1016/j.sedgeo.2010.06.017.

Lambert, L., Durlet, C., Loreau, J.P. and Marnier, G., 2006, Burial dissolution of micrite in Middle East carbonate reservoirs (Jurassic-Cretaceous): keys for recognition and timing: Marine and Petroleum Geology, v. 23, p. 79-92.

Ahr, W.M., 1989. Early diagenetic microporosity in the Cotton Valley Limestone of east Texas. Sedimentary Geology, 63(3-4), pp.275-292.

Carpenter, S.J., Lohmann, K.C., Holden, P., Walter, L.M., Huston, T.J., and Halliday, A.N., 1991, d18O values, 87Sr/86Sr and Sr/Mg ratios of Late Devonian abiotic marine calcite; implications for the composition of ancient seawater: Geochimica et Cosmochimica Acta, v. 55, p. 1991–2010.

Dravis, J.J., 1989, Deep-burial microporosity in Upper Jurassic Haynesville oolitic grainstones, east Texas: Sedimentary Geology, v. 63, p. 325–341, doi: 10.1016/0037-0738(89)90139-5.

Tavakoli, V. and Jamalian, A., 2018. Microporosity evolution in Iranian reservoirs, Dalan and Dariyan formations, the central Persian Gulf. Journal of Natural Gas Science and Engineering, 52, pp.155-165.

Dickson, J.A.D., Kenter, J.A.M., 2014. Diagenetic evolution of selected parasequences across a carbonate platform: late Paeozoic, Tengiz reservoir, Kazakhstan. J. Sediment. Res. 84 (8), 664-693.

Kaldi, J., 1989, Diagenetic microporosity (chalky porosity), Middle Devonian Kee Scarp reef complex, Norman Wells, Northwest Territories, Canada: Sedimentary Geology, v. 63, p. 241-252.

Al-Aasm, I.S. and Azmy, K.K., 1996, Diagenesis and evolution of microporosity of Middle-Upper Devonian Kee Scarp Reefs; Norman Wells; Northwest Territories; Canada: Petrographic and chemical evidence: AAPG Bulletin, v. 80, p. 82-100.

Munnecke, A., Westphal, H., Reijmer, J.J.G. and Samtleben, C.; 1997, Microspar development during early marine burial diagenesis: a comparison of Pliocene carbonates from the Bahamas with Silurian limestones from Gotland (Sweden): Sedimentology, v. 44, p. 977-990.
